# Continuous Hypobaric Hypoxia may Promote Atherosclerosis Progression in Apolipoprotein E-deficient Mice

**DOI:** 10.7150/ijms.78362

**Published:** 2023-05-08

**Authors:** Shouming Luo, Xiaogen Ma, Weiqiang Wu, Shu Lin, Mindian Li, Zhihui Zhang, Ping Zhu, Zhiyuan Song

**Affiliations:** 1 Department of Cardiovascular Medicine, Center for Circadian Metabolism and Cardiovascular Disease, Southwest Hospital, Third Military Medical University (Army Medical University), Chongqing, China.; 2 Group of Neuroendocrinology, Diabetes and Metabolism Division, Garvan Institute of Medical Research, Sydney, New South Wales, Australia.; 3 The Second Affiliated Hospital, Fujian Medical University, Quanzhou, China.

**Keywords:** altitude, atherosclerosis, hypobaric hypoxia, plaque instability, angiogenesis, inflammation

## Abstract

**Background**: Intermittent normobaric hypoxia can promote the progression of atherosclerotic plaques. However, the effect of continuous hypobaric hypoxia (CHH), which is a major feature of high-altitude environment, on atherosclerosis has not been investigated thoroughly.

**Materials and Methods**: After eight weeks of high-cholesterol diet, 30 male ApoE^-/-^ mice were randomly divided into control and CHH groups. Mice in the CHH group lived in a hypobaric chamber with an oxygen content of 10% and air pressure of 364 mmhg (equal to 5,800 m altitude above sea level) for 4 weeks, while mice in the control group lived in normoxia condition. Then all mice were euthanized and the atherosclerotic lesion size and plaque stability in the aortic root were assessed. Intraplaque angiogenesis was characterized by immunostaining of CD31 and endomucin, which are identified as specific markers of vascular endothelial cells. Immunohistochemistry and qRT-PCR were performed to measure inflammatory cytokines.

**Results**: Four weeks of CHH exposure promoted the growth of atherosclerotic lesions (*p=*0.0017) and decreased the stability of atherosclerotic plaques. In CHH group, plaque smooth muscle cells and collagen contents decreased, while plaque macrophages and lipids contents increased significantly (*p*<0.001). The contents of CD31 (*p=*0.0379) and endomucin (*p=*0.0196) in the plaque was higher in the CHH group and correlated with angiogenesis progression. Further, the content of monocyte chemotactic protein-1 (*p*=0.0376) and matrix metalloproteinase-2 was significantly higher (*p*=0.0212) in the CHH group.

**Conclusions**: CHH may accelerate atherosclerosis progression in ApoE^-/-^ mice by promoting angiogenesis and inflammation.

## Introduction

Atherosclerosis is a slowly developing inflammatory process driven by lipids and is among the most common causes of coronary artery disease and peripheral artery disease worldwide. In the late stage of atherosclerosis, the increasing lipid core and accumulated inflammatory cells make the plaque extremely unstable and prone to rupture, which often leads to acute cardiovascular events. The development of atherosclerosis is affected many factors, such as age, blood pressure, serum lipid level, blood glucose, smoking, environment, and others 1, among which hypoxia has attracted increased attention 2-5.

Intermittent hypoxia (IH) refers to the alternate state of repeated hypoxia/reoxygenation and normal oxygen cycle. Intermittent hypoxia (IH) is usually caused by specific diseases such as obstructive sleep apnea syndrome 3. Continuous hypoxia (CH) is a state of lack of oxygen for a continuous, uninterrupted period of time. There are many physiological and pathological conditions of exposure to continuous hypoxia. This may occur in a short time, such as during ischemia, or a long time of hypoxia, such as with chronic disease or residence at high altitude 6. Continuous hypoxia in high altitude areas is accompanied by low pressure, and this state is called continuous hypobaric hypoxia (CHH).

Jun et al. showed that exposure to intermittent hypoxia (IH) can accelerate the growth of atherosclerotic lesions in ApoE^-/-^ mice without affecting plaque composition 7. Intermittent hypobaric hypoxia (IHH) for 8 weeks promotes atherosclerotic plaque instability in ApoE^-/-^ mice 8. Normobaric hypoxia can promote the development of atherosclerosis in patients with sleep apnea syndrome 9. Continuous hypoxia (CH) for 3 weeks can accelerate the development of atherosclerosis in ApoE KO mice 10.

Recently, more than 10 million people have traveled to mountainous areas (altitudes > 3,000 m) for leisure or short-term work worldwide each year. The environmental factor of high altitude significantly increases the risk of Acute cardiovascular events. Epidemiological studies have shown that the incidence of sudden cardiac death is 10.9-fold higher in travelers with a history of myocardial infarction during mountain hiking, 4.7-fold higher in those with coronary heart disease but no history of myocardial infarction, and 3.4-fold higher in those with hypercholesterolemia 11. With the increase of altitude, the atmospheric pressure decreases as well as the oxygen. A previous study explored the relationship between intermittent hypobaric hypoxia (IHH) (8 hours a day for 8 weeks) and atherosclerotic plaque stability 8. However, the effect of continuous hypobaric hypoxia (CHH) on atherosclerosis in high-altitude travelers has not been fully investigated.

In order to further study the role of CHH in the development of atherosclerosis, we conducted this study: ApoE^-/-^ mice were fed a high-cholesterol diet for 8 weeks to promote the formation of atherosclerotic plaques and then exposed to a control environment or CHH for 4 weeks. The lipid profile, body weight, atherosclerotic plaque size, angiogenesis, lipid content, and inflammatory cytokines in the plaque were measured to evaluate the stability of the plaque. Therefore, our study preliminarily explored the influence of continuous hypobaric hypoxia (CHH) on the pathophysiological changes of atherosclerosis in a specific geographical environment of high altitude.

## Materials and Methods

### Experimental animals and treatments

Animal experiments were approved by the Laboratory Animal Welfare and Ethics Committee of the Third Military Medical University, China. All experiments conform to the relevant regulatory standards of the institution. Thirty male ApoE^-/-^ mice on a C57BL/6 background (6-8 weeks old, 18-22 g) were purchased from Charles River Laboratories (Beijing, China). Mice were housed in standard conditions at 22°C with a 12h/12h day/night cycle in the first week and allowed access to food and water ad libitum to adapt the environment, and then fed with a high-cholesterol diet (1.25% cholesterol and 20% fat) for the next 8 weeks to promote plaque formation. After 8 weeks of high-cholesterol diet, all mice were randomly divided into two experimental groups: control group, ApoE^-/-^ mice under normoxia conditions (n = 15); CHH group, with exposure to CHH (n = 15). Mice in the CHH group were housed in a hypobaric chamber with an oxygen content of only 10% (10% represents Fraction of inspiration O_2_ (FiO_2_)) and air pressure of 364 mmhg (approximately 0.47 atmospheric pressure, or equal to 5,800 m altitude above sea level) for 4 weeks, whereas mice in the control group were placed under normoxia conditions. During the experiment, all mice were weighed weekly. All animal experimental procedures were approved by the Army Medical University Animal Care Committee.

### Tissue preparation

After 4 weeks of CHH exposure, all mice were fasted overnight and euthanized. Blood sample was collected from the heart before saline perfusion. Serum was separated by centrifugation at 4°C. Some of the serum was used to measure the levels of triglyceride (TG), total cholesterol (TC), low-density lipoprotein (LDL), and high-density lipoprotein (HDL) by enzymatic assays at Southwest hospital and others was used for enzyme-linked immunosorbent assay (ELISA) analysis of vascular endothelial growth factor (VEGF). After isolated, some aorta trees were stored in RNAlater™ Stabilization Solution (Invitrogen, Carlsbad, CA, USA) at 4°C for total RNA extraction and the others were fixed in 4% paraformaldehyde overnight for Oil Red O (Solarbio, Beijing, China) staining. Tissue samples from the aortic roots were embedded in optimum cutting temperature compound or paraffin. Serially frozen sections (10-μm-thick) were prepared for Oil Red O and immunofluorescence staining and paraffin sections (6-μm-thick) were prepared for immunohistochemical staining.

### Histology analysis

Image-Pro Plus 6.0 software (Media Cybernetics, Rockville, MD, USA) was used for calculating the positive staining area of Oil Red O to analyze the atherosclerotic lesions of aortic trees. Serial sections were stained with hematoxylin and eosin (H&E; Solarbio) to determine plaque size in the aortic roots. Oil Red O and Masson's trichome staining (Solarbio) were performed to quantify intraplaque lipid and collagen contents, which were expressed by the percentage of the positive staining area in the total plaque area of the aortic root.

### Immunohistochemistry and immunofluorescence

To measure the intraplaque contents of smooth muscle cells, macrophages, CD31, endomucin, monocyte chemoattractant protein (MCP)-1, and matrix metalloproteinase (MMP)-2, sections were reacted with primary antibodies against α-smooth muscle actin (α-SMA, Abcam, Cambridge, UK), monocyte/macrophage (MOMA-2, Abcam), CD31 (Abcam), endomucin (Abcam), MCP-1 (Abcam), and MMP-2 (Invitrogen) at 4°C overnight. Then the sections were reacted with secondary antibodies (Zhongshan Golden Bridge Bio-technology, Beijing, China) at 37°C for 30 minutes. The intraplaque macrophages, smooth muscle cells, MMP-2, endomucin, MCP-1and CD31 contents were quantified by Image-Pro Plus 6.0 software. Atherosclerotic plaque instability index was calculated according to the standard formula: (Oil Red O^+^ area plus MOMA-2^+^area) / (α-SMA^+^ area plus collagen^+^ area) 12-13.

### Cell culture

Mouse macrophages (RAW264.7), purchased from Fenghui Biotechnology Co., Ltd, were cultured in DMEM medium (Gibco, USA), supplemented with 10% FBS (PAN, Germany) and 1% penicillin-streptomycin. Cells were cultured in hypoxia (1% O_2_) and normoxia (20% O_2_) for 24 hours.

### Quantitative real-time PCR

To measure the mRNA expression of VEGF, CD31, endomucin, MCP-1, and MMP-2 in the aorta, total RNA was extracted from the whole aorta using RNA simple Total RNA Kit (Tiangen, Beijing, China). Similarly, in order to measure the expression of *Il1α, Il1β, Il6, TNFα, Il17d, Il18* in Raw264.7 cells after hypoxia treatment, total RNA was extracted from the cells. RNA purity and concentration were measured with a spectrophotometer. Reverse transcription and cDNA synthesis were accomplished by using FastKing gDNA Dispelling RT SuperMix (Tiangen). Expression of mRNA was analyzed by qRT-PCR with CFX 96 Thermocycler (Bio-Rad,USA). All qRT-PCR experiments followed the manufacturer's instructions. The primer sequences used in this study are shown in Table 1 and Table S1, relative gene expression was calculated by using the 2^-△△CT^ method.

### Statistical analysis

All data are presented as the mean ±SEM and analyzed with GraphPad Prism-6 statistical software (La Jolla, CA, USA). When the data distributed normally and variances are equal, Student's t test was used to analyze the differences between groups. If the data did not distribute normally or the variances are uneven, nonparametric test was used. *P* < 0.05 was considered statistically significant.

## Results

### Continuous hypobaric hypoxia did not affect serum lipid profiles but caused weight loss in ApoE^-/-^ mice

**Figure 1:**
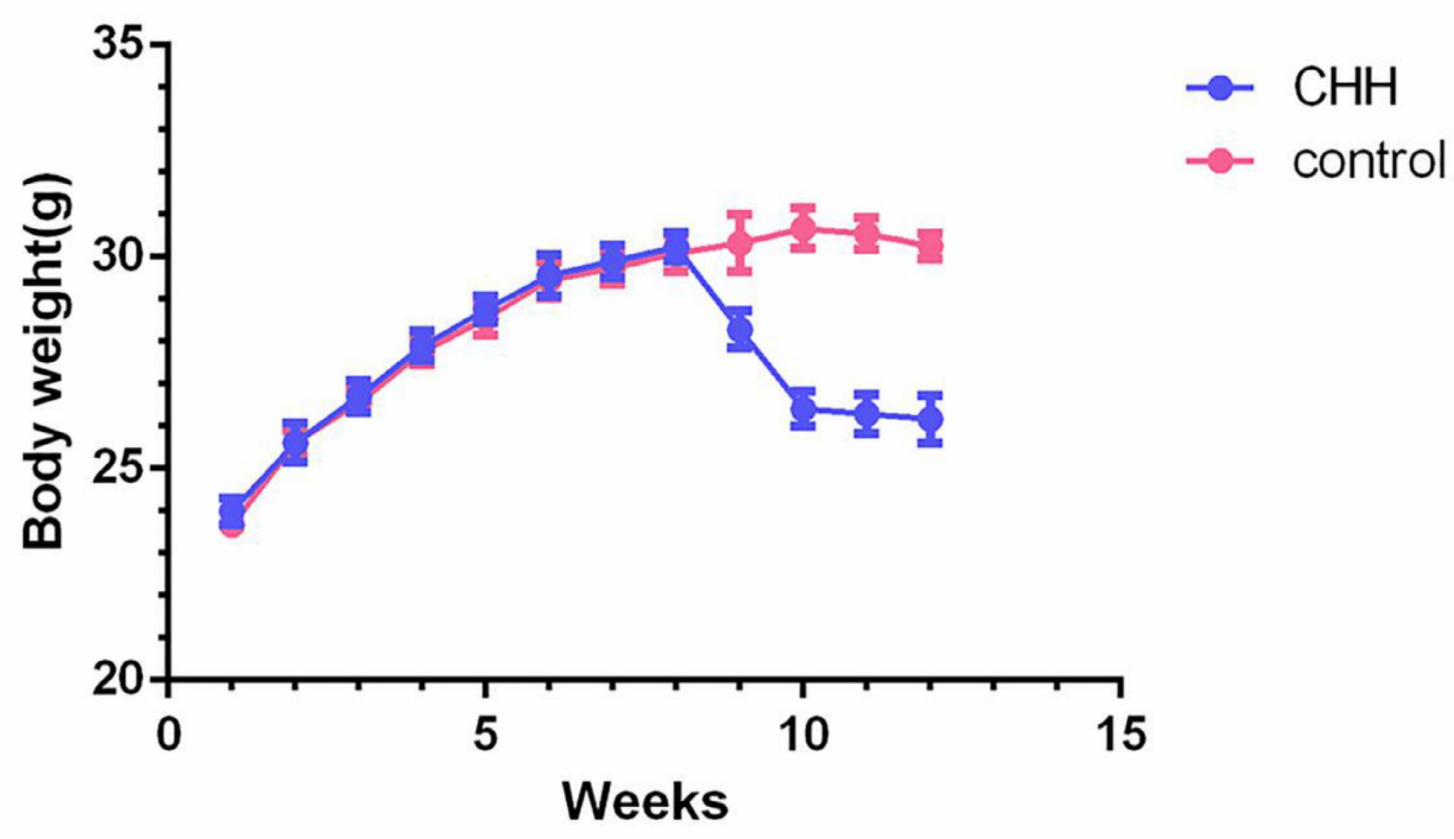
** Hypobaric hypoxia caused weight loss in ApoE^-/-^ mice.** Charts showing the weekly changes of body weight in two groups. * *P* < 0.05 vs. Control group.

Body weight and serum lipids level was compared at the end of experiment. After 4 weeks of exposure to CHH, serum levels of TC, TG, HDL and LDL did not show differences between the two groups (Table 2). The body weight of the CHH group decreased significantly compared to the control group, particularly in the first week of exposure to the decompression chamber (Figure 1).

### Continuous hypobaric hypoxia promoted atherosclerotic lesion growth in ApoE^-/-^ mice

**Figure 2:**
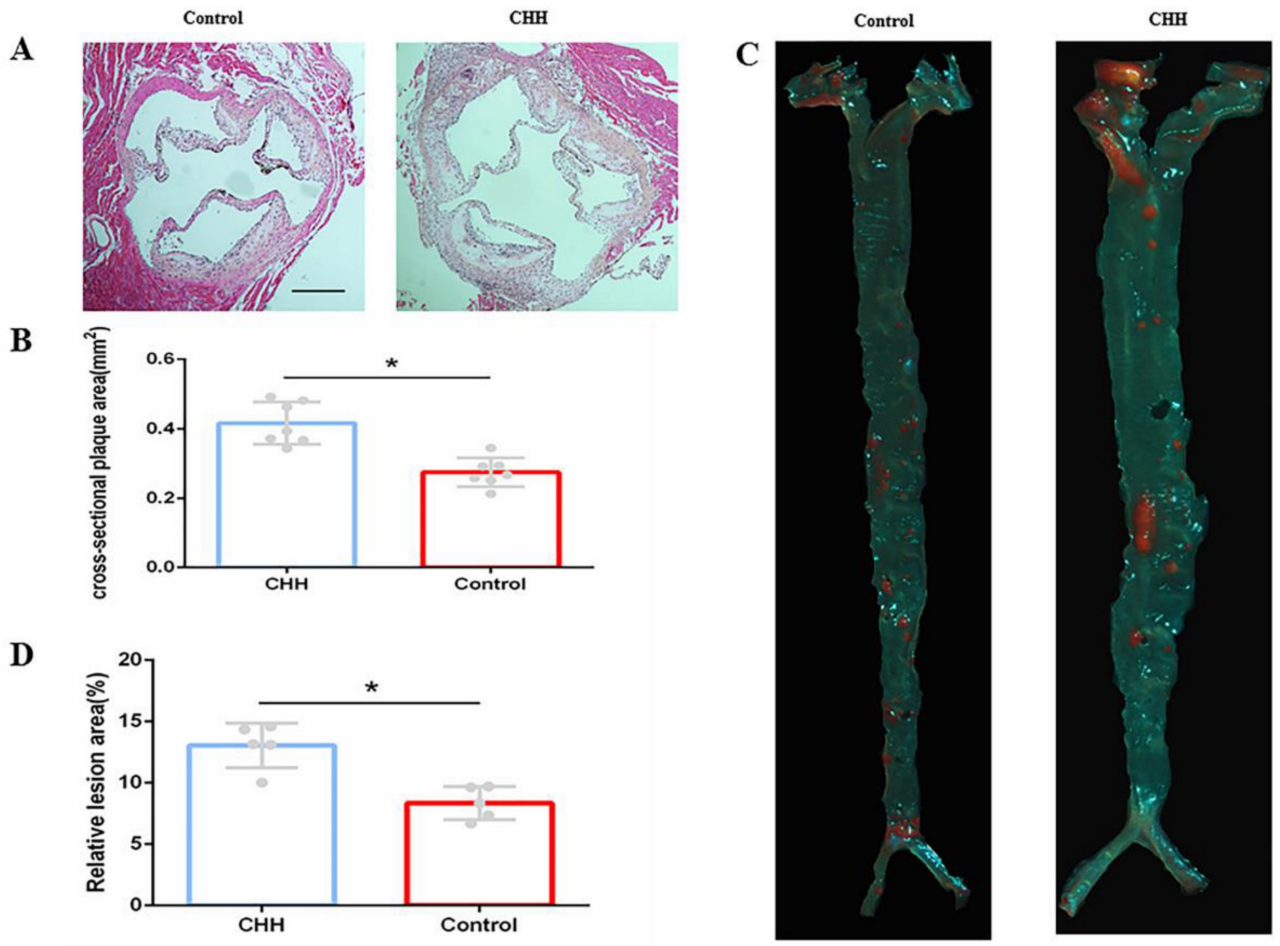
** Hypobaric hypoxia promoted atherosclerotic plaque growth in ApoE^-/-^mice.** (A) H&E staining cross-sectional plaques area; (B) Statistical data of cross-sectional plaque size in aortic roots; (C) Oil Red O staining on aortic tree in two groups of mice;(D) Statistical data of atherosclerotic lesions area of the aortic tree. * *P* < 0.05 vs. Control group. scale bar, 250μm

H&E staining showed that the size of cross-sectional plaque in the aortic root was significantly greater in the CHH group than that in the control group (Figure 2A and B). Further, Oil Red O staining of the whole aorta confirmed that exposure to CHH increased the atherosclerotic lesion area significantly ((Figure 2C and D).

### Continuous hypobaric hypoxia impaired stability of atherosclerotic plaque in ApoE ^-/-^ mice

**Figure 3:**
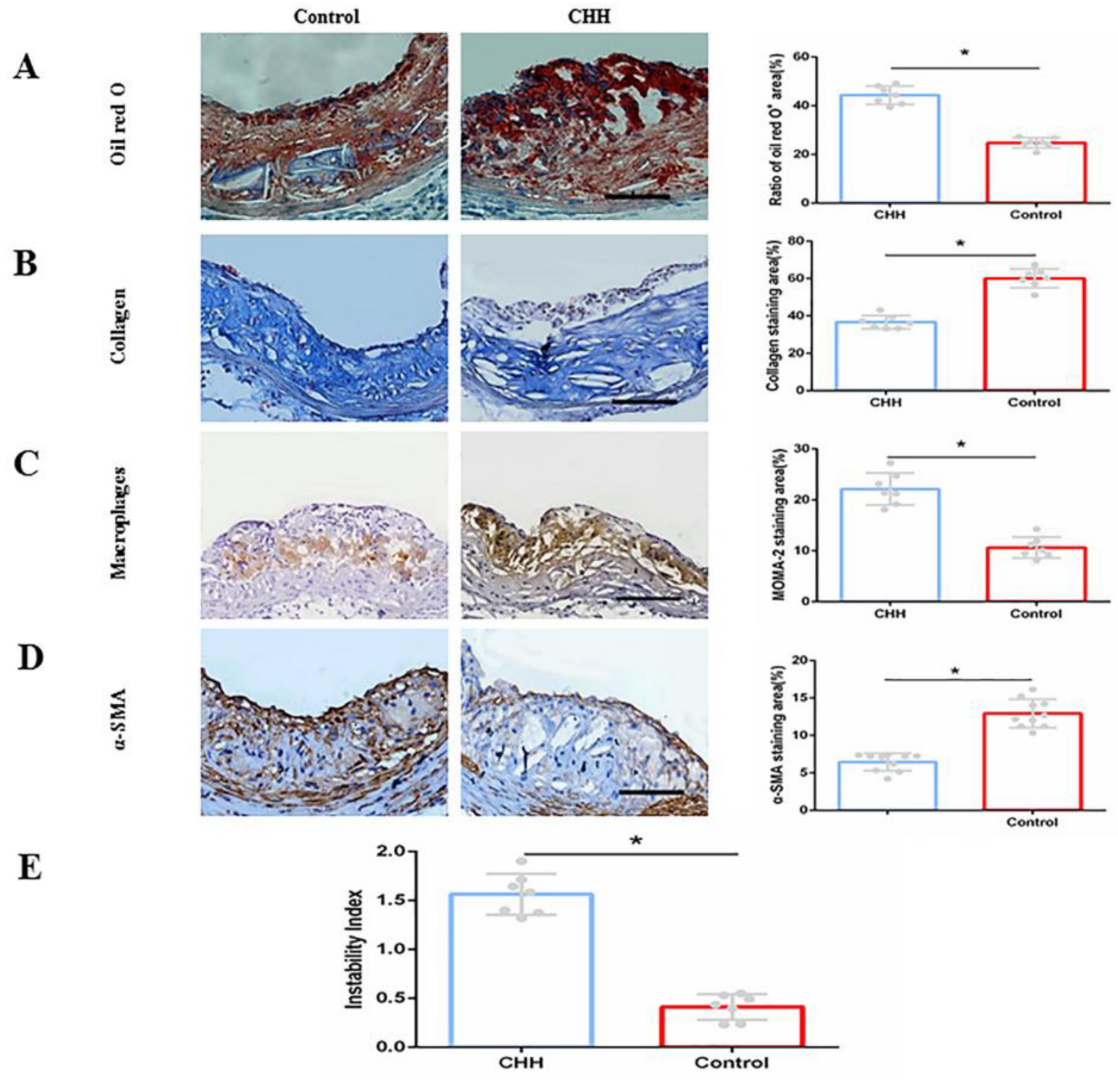
** Hypobaric hypoxia impaired stability of atherosclerotic plaque in ApoE^-/-^ mice.** (A)Oil Red O staining on intraplaque lipids and relative statistical data;(B) Masson's trichome staining on collagen and relative statistical data; (C) Immunohistochemical staining of macrophages and relative statistical data; (D) Immunohistochemical staining of smooth muscle cells and relative statistical data; (E) Plaque instability index. * P < 0.05 vs. Control group. scale bar, 100μm

To evaluate the stability of plaques, the contents of macrophages, lipids, collagen and smooth muscle cells were measured. Oil Red O staining of the aortic root demonstrated that intraplaque lipid content in the CHH group was significantly higher than that in the control group ((Figure 3A). Masson's trichome staining showed that the intraplaque collagen content (blue staining area) was significantly lower in CHH group mice than that in control mice ((Figure 3B). Furthermore, immunohistochemical analysis showed that exposure to CHH increased the intraplaque content of macrophages (MOMA-2-positive area) and reduced that of smooth muscle cells (α-SMA-positive area) compared to in the control group ((Figure 3C and D). The stability of ApoE^-/-^ mice plaque after CHH treatment was evaluated by calculated the plaque instability index. The result showed that CHH significantly increased the plaque instability index of ApoE^-/-^ mice (Figure 3E). These data suggest that exposure to CHH augments the instability of atherosclerotic plaques.

### Continuous hypobaric hypoxia promoted angiogenesis in the aorta of ApoE^-/-^ mice

**Figure 4:**
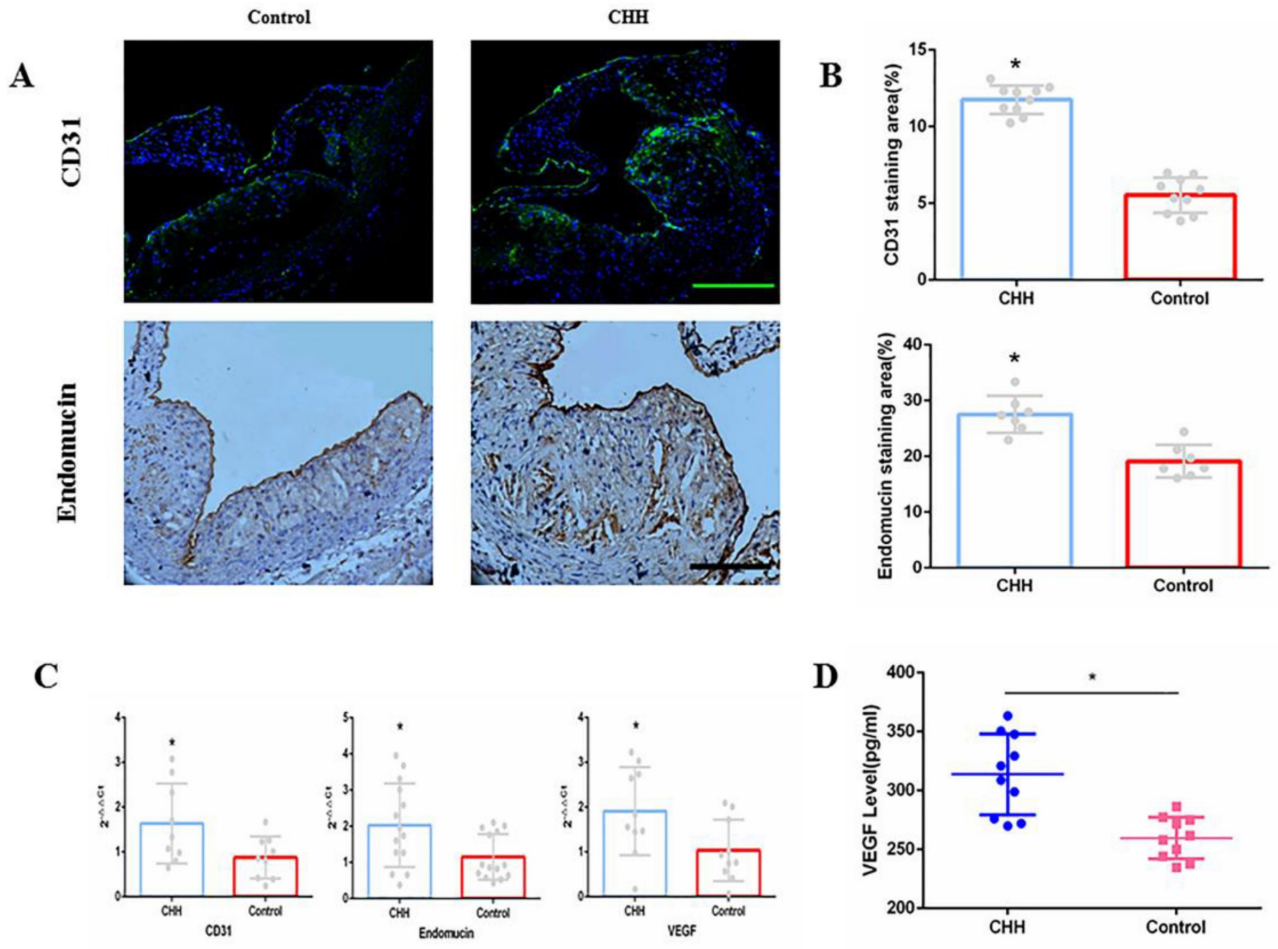
** Hypobaric hypoxia promoted expression of CD31, endomucin and VEGF in ApoE^-/-^ mice.** (A) Representative staining of CD31 and endomucin in plaques;(B) Statistical data of percentage of CD31 and endomucin in atherosclerotic lesions;(C) The mRNA expression of CD31, endomucin and VEGF in aorta;(D) Plasma VEGF level measured by ELISA. * *P* < 0.05 vs Control group; scale bar, 100μm

As markers of angiogenesis in atherosclerotic plaques, CD31 and endomucin were measured by immunofluorescence and immunohistochemistry analysis. Exposure to CHH increased the contents of CD31 and endomucin significantly (Figure 4A and B). Similarly, the qRT-PCR results showed that the CHH group exhibited significantly increased CD31 and endomucin expression in the aorta (Figure 4C). In addition, we investigated whether VEGF is involved in angiogenesis. The qRT-PCR results suggested that the mRNA expression of VEGF in the whole aorta was significantly higher in the CHH group compared to in the control group (Figure 4C). ELISA results showed that plasma VEGF level were significantly higher in the CHH group than in the control group (Figure 4D).

### Continuous hypobaric hypoxia promoted the process of inflammation in the aorta of ApoE^-/-^ mice

**Figure 5:**
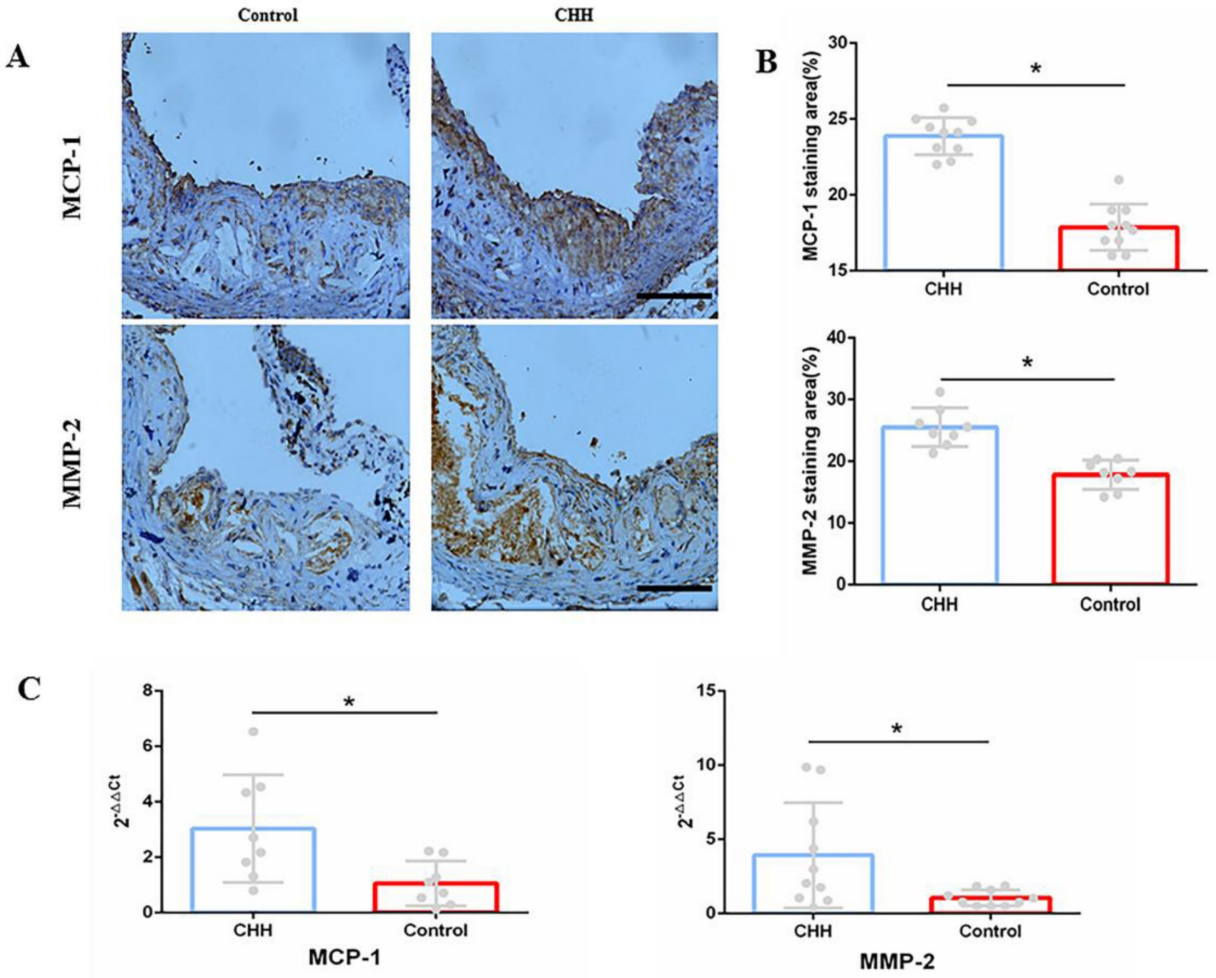
** Hypobaric hypoxia promoted expression of MCP-1 and MMP-2 in ApoE^-/-^ mice.** (A) Immunohistochemical staining of MCP-1 and MMP-2 in aortic roots; (B) Statistical data of percentage of MCP-1 and MMP-2 in aortic roots; (C) The mRNA expression of MCP-1 and MMP-2 in whole aorta. * *P* < 0.05 vs Control group; scale bar, 100μm

Macrophages are identified as one of the major contributors to intraplaque inflammation by producing inflammatory cytokines, including MCP-1 and MMP-2 14. Immunohistochemistry analysis showed that the contents of MCP-1 and MMP-2 were significantly higher in the CHH group compared to in the control group (Figure 5A and B). Further, the mRNA expression of MCP-1 and MMP-2 in the control group was lower than that in the CHH group (Figure 5C). RT-qPCR also showed that the mRNA expression of *Il1α, Il1β, Il6, TNFα, Il17d, Il18* in Raw264.7 cells were significantly increased in the hypoxia group than that in the normoxia group (Figure S1). These data support the above finding that exposure to CHH led to an increase in macrophage infiltration and a decrease in collagen content.

## Discussion

Hypoxia has been investigated as a contributing factor to atherosclerosis for several years. Chronic intermittent hypoxia exposure induces atherosclerosis in ApoE^-/-^ mice fed a high-cholesterol diet as well as a normal diet 15. Intermittent hypoxia and hypercapnia accelerated the development of atherosclerosis in ApoE^-/-^ mice 16. The aforementioned studies mainly focused on the association between normobaric hypoxia and atherosclerosis, such as the effect of obstructive sleep apnea on atherosclerosis progression 17. In recent years, an increasing number of tourists have traveled to China's Qinghai-Tibet Plateau, where the average altitude is 4,000 m above sea level and partial pressure of oxygen is only 86 mmHg, whereas this value at sea level is approximately 149 mmHg 18. After ascending to high altitude, travelers experience hypobaric hypoxia at all times, creating serious health risks, particularly in those with a history of cardiovascular disease 11. Therefore, it is very important to improve the understanding of the effects of high-altitude air conditions on atherosclerotic diseases. We exposed ApoE^-/-^ mice to CHH for 4 weeks to simulate the situation of tourists ascending from low to high altitudes. Our results demonstrated that hypobaric hypoxia is an important risk factor promoting atherosclerosis.

When exposed to high-altitude environments, body weight is often decreased; the factors primarily responsible for this effect are an increased basal metabolic rate and negative energy balance 19. The increased basal metabolic rate was greatest during the first few days of exposure to hypoxia. As the exposure time increased, sympathetic activity was attenuated and the basal metabolic rate gradually decreased 20. The negative energy balance may result from reduced energy intake caused by a loss of appetite ^[21^. This is consistent with our results: weight loss in the CHH group was extremely pronounced in the initial stage of exposure to hypoxia. As the exposure time to CHH was prolonged, the decrease in body weight was slowed and the negative energy balance was gradually corrected, indicating that we successfully simulated the environmental conditions of high altitude.

The formation and size of plaque is the most direct evidence of atherosclerosis. Nakano D et al. found that aortic atherosclerotic plaque formation was observed in ApoE^-/-^ mice with continuous normobaric hypoxia for 3 weeks (10.0 ± 0.5% O2), but no aortic atherosclerotic plaque was observed in wild type mice 10. Jiang S et al. explored the effect of intermittent hypobaric hypoxia (IHH) on aortic atherosclerosis in ApoE^-/-^ mice: 8-week ApoE^-/-^ mice were subjected to intermittent hypobaric hypoxia for 8 hours a day (simulated at 4000 m above sea level) for 8 weeks, and found that the plaque size of intermittent hypobaric hypoxia ApoE^-/-^ mice and normoxic ApoE^-/-^ mice was similar 8. In our study, ApoE^-/-^ mice after 8 weeks of high cholesterol diet were placed in a hypobaric hypoxia chamber (10% oxygen content, 364mmhg air pressure) equal to 5,800 m altitude above sea level for 4 weeks. It was found that CHH significantly increased the area of atherosclerotic plaque. It shows that IHH and CHH have different effects on promoting atherosclerotic plaque growth.

Vulnerable plaques are typically considered as non-obstructive coronary plaques, which can suddenly rupture and cause thrombosis, block the blood supply, and result in subsequent acute cardiovascular events 22. A hallmark of a vulnerable plaque is an abnormal vascular wall structure, including large lipid core areas, thin fibrous caps, infiltration of inflammatory cytokines, and degradation of collagen 23-24. Jiang S et al. found that the collagen content in the plaque of ApoE^-/-^ mice treated with IHH was significantly reduced, the expression of matrix metalloproteinase (MMP) - 9 protein was significantly increased, and the expression of tissue inhibitor of matrix metalloproteinase (TIMP) - 2 was decreased, suggesting that IHH increased the instability of the plaque 8. We found that CHH promoted enlargement of the core area of lipid deposition in the plaque of the aortic root, thinning of the fibrous cap formed by smooth muscle, increased collagen degradation and plaque instability index. Additionally, macrophage infiltration was increased significantly in the CHH group, indicating that hypobaric hypoxia aggravated plaque instability.

Intraplaque hemorrhaging plays a crucial role in atherosclerotic plaque progression and destabilization 25-27. Pathological examination revealed that intraplaque hemorrhage and plaque rupture are closely related to the intraplaque density of microvessels, and that the number of microvessels in vulnerable plaques is increased by at least 2-4-fold compared to in stable plaques 28. The formation of microvessels is a response to intraplaque hypoxia: progressive enlargement of the plaque increases the distance between the lumen and inside of the vessel wall, impairing the oxygen supply and subsequently leading to intraplaque hypoxia. During intraplaque hypoxia, the degradation of hypoxia-inducible factor (HIF)-1α is decreased, and this protein dimerizes with HIF-1ß to form an active transcription factor (HIF-1) that upregulates the expression of VEGF 29. VEGF, an essential angiogenic modulator, initiates the process of angiogenesis to compensate for the lack of oxygen supply in the plaque 30. However, these neo-microvessels are vulnerable and lack mural cells to stabilize the vessel structure, allowing extravasation of red blood cells, inflammatory cells, and red blood cell-derived cholesterol into the plaque 31. This process further promotes the progression of inflammation and destroys the stability of plaques. The main finding of this study was that CHH exposure resulted in increased VEGF transcription in plaques and promoted angiogenesis, as expressed by upregulated CD31 and endomucin expression.

In the last two decades, macrophages, the most abundant immune cells within plaques, were shown to be the major contributors to atherosclerosis and its thrombotic complications 32. In the process of atherosclerosis, monocytes in contact with activated endothelial cells are recruited into cells and differentiate into macrophages. Under hypoxic conditions, HIF-1 promotes high expression of MCP-1 in macrophages to attract more monocytes to migrate into plaques 33. In addition, HIF-1 promoted macrophages to differentiate into pro-inflammatory phenotype M1 cells and secreted pro-atherogenic cytokines and chemokines, such as tumor necrosis factor-α, interleukin-6, VEGF, and MMPs 34. A previous study demonstrated that MMP-2 and MMP-9 activity was decreased by knockdown of HIF-1α in ApoE^-/-^ mice 35. Jiang et al. also found that intermittent hypobaric hypoxia increased the expression of MMP-9 and decreased the expression of tissue inhibitor metalloproteinases-2^9^. Furthermore, Lahat et al. showed that hypoxia reduced the levels of tissue inhibitor metalloproteinases-2 in human endothelial cells 36. Taken together, increased MMP-2 levels induced by hypoxia may stimulate the degradation of collagen and consequently cause plaque instability. In the present study, the increase in plaque instability induced by CHH occurred concomitantly with increased VEGF, MMP-2, and MCP-1.

## Conclusions

In conclusion, we demonstrated that exposure of ApoE^-/-^ mice to CHH led to the enlargement of aortic atherosclerotic lesions and appearance of a greater number of neo-microvessels in the plaques. Moreover, hypoxia enhanced the activity of macrophages and promoted the accumulation of inflammatory mediators and degradation of extracellular matrix, significantly reducing plaque stability. These results indicate that hypobaric hypoxia is an important risk factor for cardiovascular events at high altitudes.

## Supplementary Material

Supplementary materials and methods, figure and table.

## Figures and Tables

**Table 1:** qRT-PCR primers sequences

Primers	Sequence 5'-3'
**CD31**	Forward: CACAACAAACAAGCTAGCAAGA Reverse: TTTGGCTGCAACTATTAAGGTG
**Endomucin**	Forward: ACCATGTCACTGCTTCAAGATA Reverse: GTCTCTCTTCCAGCATATTCGA
**VEGF**	Forward: TAGAGTACATCTTCAAGCCGTC Reverse: CTTTCTTTGGTCTGCATTCACA
**MCP-1**	Forward: TTAAAAACCTGGATCGGAACCAA Reverse: GCATTAGCTTCAGATTTACGGGT
**MMP-2**	Forward: ACTTTGAGAAGGATGGCAAGTA Reverse: CTTCTTATCCCGGTCATAGTCC
**β-Actin**	Forward: GTGCTATGTTGCTCTAGACTTCG Reverse: ATGCCACAGGATTCCATACC

VEGF, vascular endothelial growth factor; MCP-1, monocyte chemotactic protein-1; MMP2, matrix metalloproteinase-2.

**Table 2:** Serum lipid level

Groups	TG (mmol/L)	TC (mmol/L)	HDL (mmol/L)	LDL-C (mmol/L)
CHH	0.62±0.03	9.570±0.48	0.85±0.04	7.73±0.35
Control	0.67±0.06	10.03±0.80	0.95±0.06	8.24±0.51
*P*	ns	ns	ns	ns

TG: triglyceride;TC: total cholesterol; HDL: high-density lipoprotein; LDL: low-density lipoprotein; ns: not significant.
