# Supplementary material for: Continuous Hypobaric Hypoxia may Promote Atherosclerosis Progression in Apolipoprotein E-deficient Mice
Source: Int J Med Sci. 2023 May 8;20(7):849–57. doi: 10.7150/ijms.78362 (PMC10266041; doi:10.7150/ijms.78362)
Supplement: Supplementary file 1 — Supplementary materials and methods, figure and table. [file ijmsv20p0849s1.pdf]

Figure S1

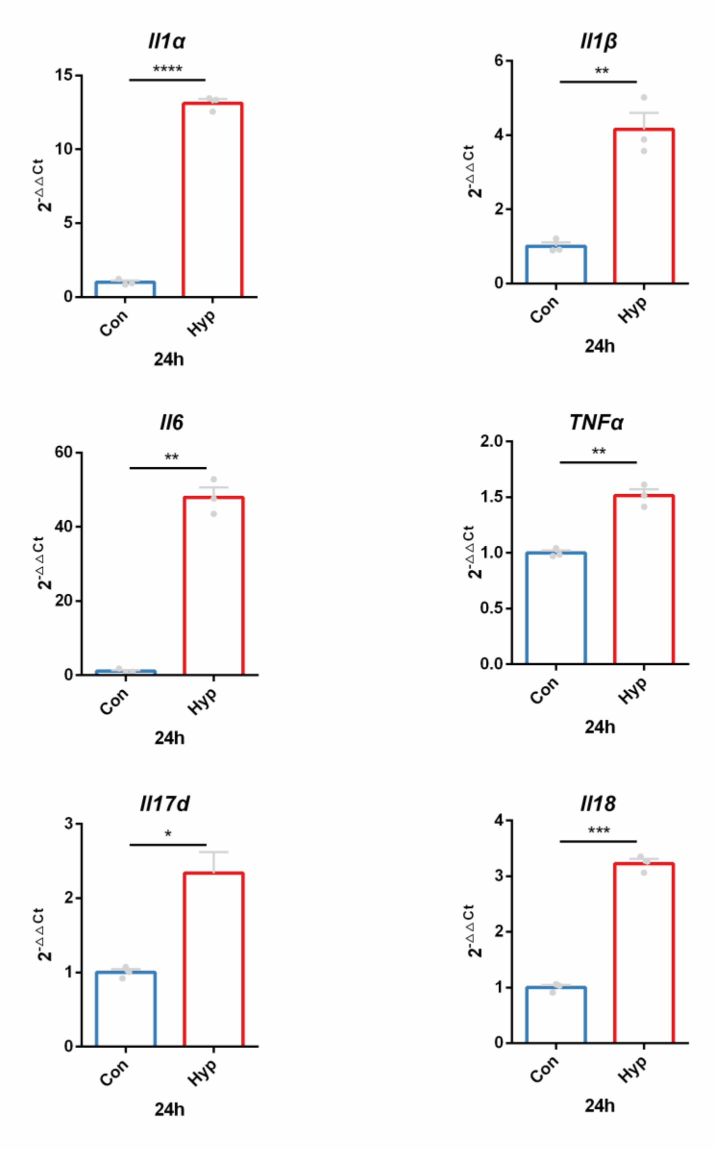

Figure S1.Hypoxia promotes the expression of inflammatory factors in macrophages. Data were presented as mean ± SEM, unpaired t-test or Welch's t test, *$P < 0.05$, ** $p < 0.01$, *** $p < 0.001$, **** $p < 0.0001$.

Table S1

Table S1. Primer Sequence Used in RT-qPCR Analysis

Gene	Sequence 5′ -3′
**Il1α**	Forward: CGAAGACTACAGTTCTGCCATT
	Reverse: GACGTTTCAGAGGTTCTCAGAG
**Il1β**	Forward: GCAACTGTTCCTGAACTCAACT
	Reverse: ATCTTTTGGGGTCCGTCAACT
**IL6**	Forward: TAGTCCTTCCTACCCCAATTTCC
	Reverse: TTGGTCCTTAGCCACTCCTTC
**TNFα**	Forward: CTGGATGTCAATCAACAATGGGA
	Reverse: ACTAGGGTGTGAGTGTTTTCTGT
**Il17d**	Forward: AGCACACCCGTCTTCTCTC
	Reverse: GCTGGAGTTCGCACTGTCC
**Il18**	Forward: GACTCTTGCGTCAACTTCAAGG
	Reverse: CAGGCTGTCTTTTGTCAACGA
**β-Actin**	Forward: GTGCTATGTTGCTCTAGACTTCG
	Reverse: ATGCCACAGGATTCCATACC

Materials and Methods

Cell culture

Mouse macrophages (RAW264.7), purchased from Fenghui Biotechnology Co., Ltd, were cultured in DMEM medium (Gibco, USA), supplemented with 10% FBS (PAN, Germany) and 1% penicillin–streptomycin. Cells were cultured in hypoxia (1% O_2) and normoxia (20% O_2) for 24 hours.

Quantitative real-time PCR

To measure the expression of *Il1α, Il1β, Il6, TNFα, Il17d, Il18* in Raw264.7 cells after hypoxia treatment, total RNA was extracted from the cells (Tiangen, Beijing, China). RNA purity and concentration were measured with a spectrophotometer. Reverse transcription and cDNA synthesis were accomplished by using FastKing gDNA Dispelling RT SuperMix (Tiangen, Beijing, China). Expression of mRNA was analyzed by qRT-PCR with CFX 96 Thermocycler (Bio-Rad, USA). All qRT-PCR experiments followed the manufacturer's instructions. The primer sequences used in this study are shown in Table S1, relative gene expression was calculated by using the $2^{-\Delta\Delta CT}$ method.
